# Ecological analysis of demographic-, nutritional- and housing-related factors associated with anaemia among women of reproductive age group in Nigeria

**DOI:** 10.1186/s41043-022-00334-6

**Published:** 2022-12-09

**Authors:** Yusuf Olushola Kareem, Edward K. Ameyaw, Oluwasomidoyin O. Bello, Rukiyat A. Abdus-Salam, Olatunji O. Lawal, Gbolahan Obajimi, Yussuf Kofoworola Alade, Imran O. Morhason-Bello

**Affiliations:** 1 United Nation Population Fund, Nigeria Country Office, Abuja, Nigeria; 2 Institute of Policy Studies and School of Graduate Studies, Lingnan University, Tuen Mun, New Territories Hong Kong; 3 Department of Obstetrics and Gynaecology, Faculty of Clinical Sciences, College of Medicine, University of Ibadan, Ibadan, Nigeria; 4 Paelon Memorial Hospital, Victoria Island, Lagos, Nigeria; 5 Institute for Advanced Medical Research and Training, College of Medicine, University of Ibadan, Ibadan, Nigeria

## Abstract

**Introduction:**

Anaemia remains a major public health concern, particularly, in sub-Saharan Africa (SSA), where it is one of the causes of maternal death. The most common cause of maternal anaemia is iron deficiency or malnutrition. This study examined the prevalence of and risk factors for anaemia among women that participated in the Nigerian Demographic Health Survey.

**Method:**

We used data of 14,454 women that participated in the 2018 Nigeria Demographic and Health Survey (NDHS). We extracted information such as demographic, social and housing, dietary characteristics and haemoglobin concentration. The descriptive statistic results, prevalence and 95% confidence interval (CIs) of anaemia with the selected respondents background characteristics were presented. The Poisson regression model with robust variance was used to assess the risk of anaemia among women of reproductive age. All analyses were weighted and adjusted for the complex survey design. Statistical significance was interpreted at p value < 0.05.

**Results:**

Maternal status, body mass index, education, residence, religion, ethnicity, region and type of cooking fuel were all important determinants of anaemia. The prevalence of anaemia was high among pregnant women (61.8%; 95% CI: 58.5–65.0), adolescents (60.4%; 95% CI: 58.1–62.6), underweight women (62.6%; 95% CI: 59.5–65.5), women who had no formal education (64.1%; 95% CI: 62.2–66.0) and those who belonged to the poorest wealth quintile (65.8%; 95% CI: 63.1–68.4). Similarly, anaemia was high among women residing in rural areas (61.5%; 95% CI: 60.0–63.0), Muslims (59.9%; 95% CI: 58.1–61.6) and women with six or more children (62.1%; 95% CI: 60.0–64.1). The risk of anaemia were 2% less likely among women who took minimum adequate diet compared to those who do not.

**Conclusion:**

To date, this is the largest data on maternal anaemia in Nigeria. The study highlighted the high burden of maternal anaemia in the country and different risk factors (medical and social) that are associated with this medical condition among women of reproductive age. We recommend future longitudinal studies to test hypothesis in order to assess whether there is any causal relationship between identified risk factors and anaemia in this group of women.

**Supplementary Information:**

The online version contains supplementary material available at 10.1186/s41043-022-00334-6.

## Introduction

Anaemia remains a major public health concern that currently affects nearly one-third of the world’s population [1]. The most common cause of anaemia is due to iron deficiency; it may be caused by inadequate dietary intake or malabsorption. The highest burden of anaemia is in South Asia, Central and West Africa affecting over 1.6 billion people [2, 3].

In 2012 and 2013, the World Health Organization (WHO) facilitated a global resolution at the World Health Assembly on strategies to measure progress and assess global interventions on nutrition- and diet-related non-communicable diseases, which eventually form a key target of Sustainable Development Goals for eradicating all form of malnutrition by 2030 [4, 5]. In 2013, 183,000 deaths were attributed to iron deficiency anaemia worldwide; a higher risk was recorded among children relative to adults and a higher risk in women compared to men[1, 6]. In the same estimates, pregnant and lactating women had a higher risk of anaemia compared to non-pregnant women.

The WHO classified anaemia in pregnancy based on the assessment of haemoglobin concentration. Anaemia was defined as haemoglobin of less than 110 g/l (11 g/dl) in pregnant women, while anaemia in non-pregnant women was defined as haemoglobin concentration of less than 120 g/l (12 g/dl) and haemoglobin estimation below 130 g/l (13 g/dl) men [7]. According to the WHO Global Health Observatory, one in three women of reproductive age is anaemic [7]. In the same report, more than half of women in most countries in the sub-Saharan Africa region were anaemic, with no significant change in the last decade [7]. In Nigeria, the burden of maternal anaemia has remained high for many years despite interventions to prevent and treat this condition [8]. For example, the prevalence of anaemia among women of reproductive age was 55.3% (95% UI: 42.6–68.2) in 2010 and remain stagnant at 55% (95% UI: 43.7–65) in 2018 [7]. Anaemia is one of the major causes of maternal death in Nigeria accounting for more than 10% of pregnancy-related deaths [9, 10].

Generally, anaemia in pregnancy is associated with adverse pregnancy outcomes such as recurrent pregnancy loss, preterm labour, intrauterine growth restriction, unexplained foetal death and poor maternal reserve to withstand bleeding after childbirth amongst other potential complications [11, 12]. In a non-pregnant state, an anaemic woman could suffer from heart failure, persistent generalized weakness and loss of time to attend to daily demands [13]. Evidence abounds that anaemia in women is associated with poor nutrition, poverty, taboos against certain diet, sociocultural practice, certain obstetric factors—teenage pregnancy and short birth intervals, haemoglobinopathy and living in some disease endemic areas such malaria and worm infestation [9, 14, 15]. Studies on the risk factor assessment for anaemia have largely focused on clinical risk factors in Nigeria, mostly in health facility settings, and there are no known studies that explored these risk factors using population-level data [16–18].

There are several interventions designed to prevent anaemia, particularly, in low-middle income countries. For example, counselling on balanced nutrition, routine iron supplementation in pregnancy and treatment of important causative organisms such as malaria and worm infestation have been adopted and integrated into routine maternal healthcare in many countries in sub-Saharan Africa including Nigeria. Despite these investments, the WHO report still showed that the global target of 50% reduction in maternal anaemia is unlikely to be achieved by the target date of 2025 [19].

Although anaemia remains a common morbidity; unfortunately, there are limited compelling nationally representative data, especially in Nigeria that examined pattern of anaemia in different categories of women and how key factors such as diet and other social conditions affected the prevalence. These data are needed in order for policy makers and programmers to plan context specific interventions to mitigate the burden of anaemia among women. The study investigated the prevalence of anaemia and key drivers of anaemia among women of reproductive age, categorized as pregnant, breastfeeding and nether pregnant nor breastfeeding, that participated in the 2018 Nigeria Demographic Health Survey (NDHS).

## Materials and methods

### Data source and sampling strategy

We used data of women that participated in the 2018 NDHS. The Demographic and Health Survey (DHS) is a cross-sectional survey that adopts a two-stage stratified design to produce reliable estimate for basic population demographics and health indicators. This sampling design allows for appropriate estimation at the national, regional and state level, as well as urban and rural areas. The 36 states and the federal capital territory (FCT) of Nigeria were stratified into urban and rural areas, making a total of 74 sampling strata. The first stage used a probability proportional to Enumeration Areas (EAs) to select 1,400 Enumeration Areas sizes (EAs) using the sampling frame of the 2006 National Population and Housing Census (NPHC). In the second stage, 30 households were selected in every EAs by an equal probability systematic sampling. A total of 42,121 women of reproductive age (15–49 years) from 41,668 households were selected for interview and the response rate was 99% at both the household and individual level. The detail description of sampling methods and implementation is available at the DHS website and in the 2018 NDHS report [20]. The survey protocol was reviewed by ICF Institutional Review Board and the National Health Research Ethics Committee of Nigeria. The DHS maintains strict adherence to confidentiality. The datasets used for this study can be accessed from https://dhsprogram.com/data/dataset/Nigeria_Standard-DHS_2018.cfm for free after seeking permission from the DHS Program.

### Description of variables

#### Outcome variable

The outcome variable was presence of anaemia or not among women of reproductive age. An on-site haemoglobin analysis was done using a HemoCue®Hb 201 + device by collecting blood through a finger prick and then drawn into a microcuvette. The results of the test were provided both verbally and in writing. The classification of anaemia was based on the WHO’s haemoglobin threshold [6]. Anaemia was defined as haemoglobin level < 11.0 g/dl among pregnant women or haemoglobin level of < 12.0 g/dl among others including breastfeeding women. Pregnant women with haemoglobin level below 7 g per decilitre (g/dl) and breastfeeding or non-pregnant women with haemoglobin level below 8 g/dl were referred to a health care facility. In this study, women were classified as pregnant, breastfeeding and neither pregnant nor lactating. Women who were both pregnant and breastfeeding were less than one percent and were excluded from this study.

#### Explanatory variables

The explanatory variables were categorized into 3 blocks; the first block measures the dietary consumption characteristics. The dietary consumption was further classified as minimal adequate dietary diversity if a woman takes at least 5 out of the 10 food groups: (1) grains, white tubers and roots, (2) legumes, (3) nuts and seeds, (4) milk, cheese and yoghurt, (5) meat, fish and poultry, (6) eggs, (7) dark green leafy vegetables, (8) fruits and vegetables rich in vitamin A, (9) other vegetables and (10) other fruits in the last 24 h preceding the survey [20, 21]. The second block involves some selected sociodemographic characteristics: maternal status (pregnant, breastfeeding and others), age group in 5 year interval (15–19, 20–24, 25–29, 30–34, 35–39, 40–44 and 45–49), body mass index (categorized as underweight, < 18.5; normal 18.5–24.9; overweight, 25.0–29.9; and obese, ≥ 30.0), parity (none, 1, 2–3, 4–5 and 6 or more), level of education (none, primary, secondary and higher), wealth quintile (poorest, poor, middle, richer and richest), place of residence (urban and rural), religion (catholic, other Christians, Islam and others), ethnicity (Fulani, Hausa, Igbo, Yoruba and other ethnic minorities), marital status (never married, currently married and formerly in union), slept under insecticide treated net the last night preceding the survey (yes vs no), smoke cigarette (yes vs no) and respondent stature which was computed using two standard deviation (SD) from the population median height and categorized as short, < -2SD; normal, -2SD to 2SD; and tall, > 2SD. The third block includes the social/housing condition variables which consist of type of cooking fuel (clean and polluted), and source of water, type of toilet facilities and housing materials which comprised floor, wall and roof materials were classified as unimproved or improved. The cooking fuel was categorized as clean or modern fuel if the sources of cooking were electricity, liquefied petroleum as well as natural gas. It was polluted if the sources were from kerosene or solid fuels including coal, lignite charcoal, wood, straw/grass, agricultural crop and animal waste [22]. Categorization of water sources followed the WHO/UNICEF Joint Monitoring Programme (JMP) definition: thus, unimproved if water were from unprotected well, unprotected spring and surface water such as river, dam, pond, stream, canal or irrigation. However, public tap or standpipe, tube well or borehole, protected well, protected spring, rainwater, tanker truck and cart with small tank, bottled, sachet and water piped into dwelling, yard, plot or piped into the neighbourhood were classified as water from improved sources [23].The type of sanitary facility was also classified based on the WHO/UNICEF Joint Monitoring Programme (JMP). Sanitation facilities classified as unimproved were pit latrine without slab or open pit, bucket toilet, hanging toilet or latrine, toilet that flushes to somewhere else, and those who had no toilet facilities and practice either in the bush or field. Improved facilities included sanitary system that flush to piped sewer system, septic tank, flush to pit latrine, flush to unknown location, ventilated improved pit, pit latrine with slab and composting toilet [23, 24].

We included housing condition as an explanatory variable in our analysis. Although, this indicator reflects the socio-economic status of respondents. An earlier study conducted in Nigeria had found an association between housing condition and malaria, which plays a major causative role for anaemia and are usually associated [7, 25, 26]. The housing materials were classified as unimproved if none of the floor, roof and wall materials were made from improved materials and classified as partially improved if at least one but not all of these materials were improved and as totally improved if all of these materials were classified as improved [25]. The floor materials classified as unimproved were earth, sand, dung, rudimentary, wood planks, palm bamboo while cement, ceramic tiles, vinyl asphalt strips, parquet and polished wood. For the wall, materials made from cane, palm or trunks, dirt, rudimentary, bamboo with mud, stone with mud, uncovered adobe, plywood and houses with no wall were classified as unimproved, while cement, stone with lime or cement, cement blocks and bricks were improved materials. Roofing materials were classified as unimproved if there were no roof, thatch or palm leaf, sod, rudimentary, rustic mat, palm or bamboo, wood planks, wood or other materials. However, roof made with cement or roofing shingles was classified as improved roofing materials.

#### Statistical analysis

We used data of women (15–49 years) who had result of haemoglobin estimation and had complete responses on all other covariates considered for this analysis. The descriptive summaries, prevalence and 95% confidence intervals (CIs) of anaemia by respondent characteristics were computed. In order to determine the likelihood of anaemia among women of reproductive age, the Poisson regression model with robust variance was used to assess the relative risk of anaemia by dietary intake while adjusting for other respondents’ characteristics. We used the robust Poisson regression and not the logistic regression model because the prevalence of anaemia was high (> 10%) and we intend to interpret our results in terms of risk ratios and not odds ratios. The robust Poisson model was utilized due to the non-convergence of the log-binomial model [27–30]. First, a crude analysis was performed to determine association between anaemia and sociodemographic, dietary and housing characteristics. With the exception of age, all explanatory variables that were not significant at p < 0.10 were excluded from the adjusted model. In the adjusted model, we used hierarchical modelling in three steps to test the association between the risk of anaemia and the explanatory variable. The first step involves only the dietary characteristics of respondents. In the second step, we added sociodemographic variables to the model, while the final step involves addition of social/housing condition characteristics. A multicollinearity test was performed to check for possible collinearity using a variance inflation factor cut-off of five. All analyses were weighted, adjusted for the complex survey design, and were performed using Stata 15.0 (StataCorp LLC, College Station, Texas, USA). Statistical significance was interpreted at p value < 0.05.

## Results

### Sociodemographic variables

A total of 14,454 women were eligible for this study, and 3,713 (25.7%) of them were breastfeeding, while 1,469 (10.2%) were currently pregnant (Table 1). About one in five respondents were adolescents, 11.5% were underweight, and 29.2% were either overweight or obese. Almost one in four of women had no child, 22.7% were never in a marital union, and 33.1% had no formal education, while 35% belonged to either the poorest or poorer wealth quintiles. Women who reside in the rural areas were (53.6%) more than those in the urban areas. About a quarter of the respondents were from the north-western region, and more than half (52.1%) of women did not sleep under the insecticide treated net (ITN).

**Table 1:** Baseline characteristics, prevalence and crude risk ratio of women of characteristics, NDHS 2018 (N = 14,454)

Variables	Frequency (%)	Prevalence, % (95% CI) p value	Crude PR (95% CI) p value
*Women status*		p < 0.05	p < 0.05
Non-pregnant and non-lactating	9272 (64.2%)	56.6 (55.1–58.0)	Reference
Pregnant	1469 (10.2%)	61.8 (58.5–65.0)	1.03 (1.01–1.06)**
Breastfeeding	3713 (25.7%)	59.4 (57.5–61.4)	1.02 (1.00–1.03)**
Age		p < 0.106	p < 0.137
15–19	2695 (18.7%)	60.4 (58.1–62.6)	Reference
20–24	2253 (15.6%)	56.0 (53.4–58.7)	0.97 (0.95–0.99)**
25–29	2608 (18.0%)	55.8 (53.2–58.4)	0.97 (0.95–0.99)**
30–34	2286 (15.8%)	58.0 (55.4–60.5)	0.99 (0.97–1.01)
35–39	2062 (14.3%)	59.1 (56.3–61.9)	0.99 (0.97–1.01)
40–44	1312 (9.1%)	57.5 (54.3–60.6)	0.98 (0.96–1.01)
45–49	1239 (8.6%)	57.7 (54.4–60.9)	0.98 (0.96–1.01)
*BMI*		p < 0.001	p < 0.001
< 18.5 (Too thin for height)	1662 (11.5%)	62.6 (59.5–65.5)	1.02 (0.99–1.04)
18.5–24.9 (Normal)	8724 (60.4%)	60.2 (58.6–61.7)	Reference
25.0–29.9 (Overweight)	2643 (18.3%)	53.2 (50.7–55.6)	0.96 (0.94–0.97)***
≥ 30.0 (Obese)	1425 (9.9%)	46.8 (43.6–50.0)	0.92 (0.89–0.94)***
*Stature*		p < 0.627	p < 0.594
Short (< -2SD)	277 (1.9%)	59.6 (52.2–66.6)	1.01 (0.97–1.06)
Normal (-2SD—2SD)	13,886 (96.1%)	57.7 (56.5–58.9)	Reference
Tall (> 2SD)	291 (2.0%)	57.8 (56.7–59.0)	1.02 (0.98–1.06)
*Parity*		p < 0.001	p < 0.001
None	3615 (25.0%)	56.8 (54.7–58.9)	Reference
1	1708 (11.8%)	57.6 (54.6–60.5)	1.00 (0.98–1.03)
2–3	3360 (23.3%)	55.7 (53.6–57.8)	0.99 (0.97–1.01)
4–5	2708 (18.7%)	57.2 (54.9–59.5)	1.00 (0.98–1.02)
≥ 6	3063 (21.2%)	62.1 (60.0–64.1)	1.03 (1.02–1.05)
*Marital status*		p = 0.145	p = 0.162
Never in union	3277 (22.7%)	56.5 (54.2–58.8)	Reference
Currently in union	10,434 (72.2%)	58.4 (57.1–59.7)	1.01 (1.00–1.03)
Formerly in union	742 (5.1%)	55.4 (51.3–59.6)	0.99 (0.96–1.02)
*Highest level of education*		p < 0.001	p < 0.001
No education	4788 (33.1%)	64.1 (62.2–66.0)	Reference
Primary	2214 (15.3%)	58.7 (56.2–61.0)	0.97 (0.95–0.98)***
Secondary	5958 (41.2%)	47.3 (43.9–50.8)	0.95 (0.93–0.96)***
Tertiary	1495 (10.3%)	57.8 (56.7–59.0)	0.90 (0.87–0.92)***
*Wealth quintile*		p < 0.001	p < 0.001
Poorest	2331 (16.1%)	65.8 (63.1–68.4)	Reference
Poorer	2725 (18.9%)	61.7 (59.1–64.2)	0.98 (0.95–0.99)**
Middle	2952 (20.4%)	59.2 (56.9–61.4)	0.96 (0.94–0.98)***
Richer	3170 (21.9%)	55.3 (53.1–57.6)	0.94 (0.92–0.96)***
Richest	3277 (22.7%)	50.2 (47.7–52.7)	0.91 (0.89–0.93)***
*Residence*		p < 0.001	p < 0.001
Urban	6706 (46.4)	53.6 (51.8–55.4)	Reference
Rural	7748 (53.6)	61.5 (60.0–63.0)	1.05 (1.04–1.07)***
*Religion*		p < 0.001	p < 0.001
Catholic	1525 (10.6%)	56.5 (53.6–59.4)	Reference
Other Christian	5376 (37.2%)	55.6 (54.0–57.2)	0.99 (0.97–1.01)
Islam	7468 (51.7%)	59.9 (58.1–61.6)	1.02 (1.00–1.04)**
Other religion	85 (0.6%)	47.3 (35.5–59.4)	0.95 (0.87–1.03)
*Ethnicity*		p < 0.001	p < 0.001
Fulani	821 (5.68%)	64.2 (59.9–68.4)	Reference
Hausa	4008 (27.7%	61.0 (58.6–63.4)	0.98 (0.95–1.01)
Igbo	2255 (15.6%)	64.7 (62.1–67.2)	1.00 (0.97–1.03)
Yoruba	2409 (16.7%)	51.4 (48.7–54.1)	0.92 (0.89–0.95)***
Other ethnic minorities	4962 (34.3%)	54.2 (52.3–56.1)	0.94 (0.91–0.97)***
*Regions*		p < 0.001	p < 0.001
North Central	2080 (14.4%)	55.1 (52.4–57.7)	Reference
North-East	2189 (15.2%)	58.3 (55.4–61.3)	1.02 (1.00–1.05)
North-West	3889 (26.9%)	58.9 (56.4–61.4)	1.02 (1.00–1.05)**
South-East	1769 (12.2%)	65.9 (63.1–68.5)	1.07 (1.04–1.10)***
South–South	1791 (12.4%)	60.4 (57.1–63.5)	1.03 (1.01–1.06)**
South-West	2735 (18.9%)	51.2 (48.5–53.9)	0.98 (0.95–1.00)**
*Slept under ITN*		p = 0.245	p = 0.245
No	7531 (52.1%)	57.2 (55.7–58.8)	Reference
Yes	6923 (47.9%)	58.5 (56.9–60.1)	1.01 (0.99–1.02)
*Smoke cigarettes*		p = 0.664	p = 0.674
No	14,429 (99.8)	57.8 (56.7–59.0)	Reference
Yes	25 (0.2%)	53.3 (33.2–72.4)	0.97 (0.85–1.11)
Total	14,454 (100%)	57.8 (56.7–59.0)	

*p value < 0.1, **p value < 0.05, ***p value < 0.01

### Prevalence and risk factors for anaemia

**Fig. 1:**
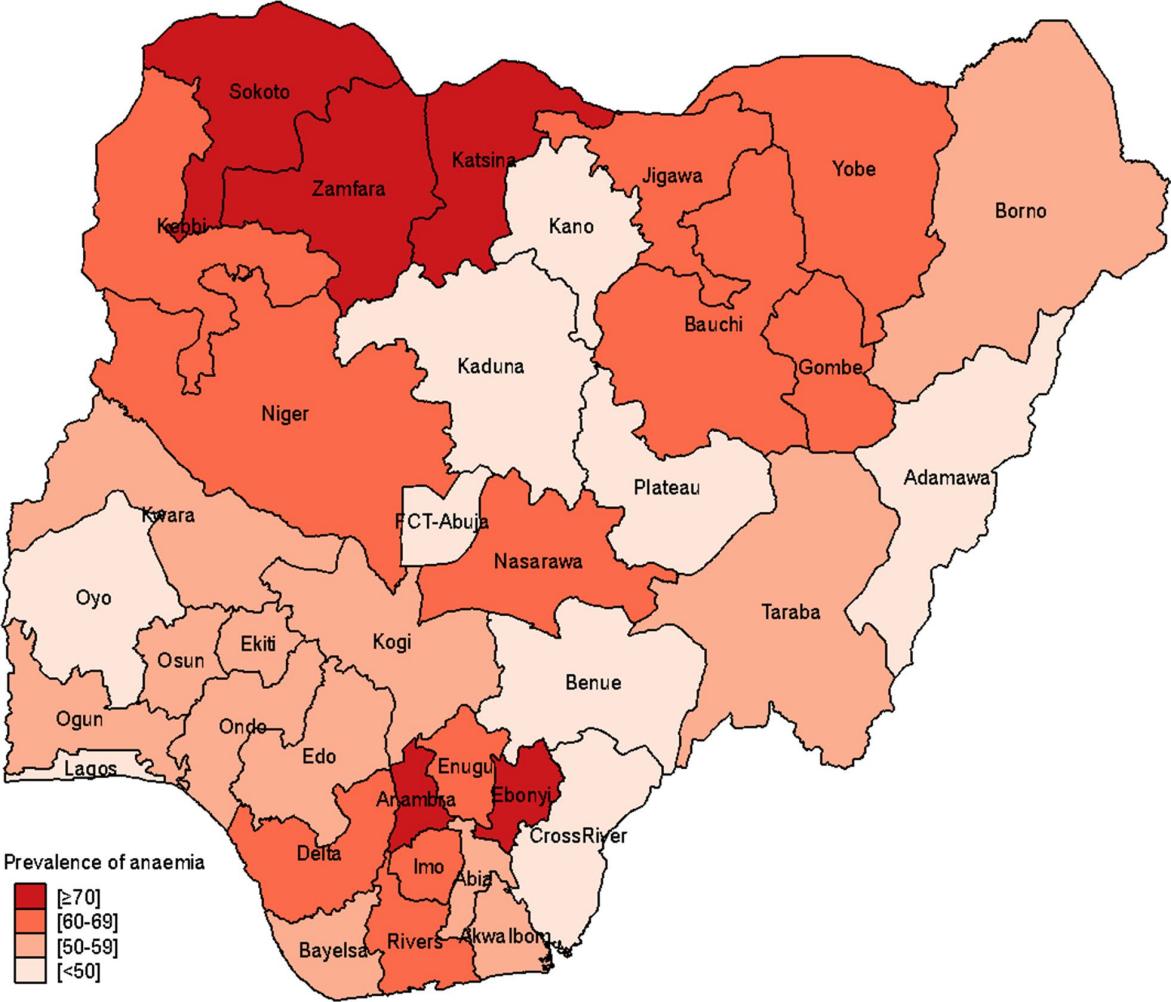
The prevalence of anaemia among women of reproductive age by States

Table 1 shows the prevalence of anaemia by the characteristics of respondents. The prevalence of anaemia were high among these groups: pregnant women (61.8%; 95% CI: 58.5–65.0), adolescents (60.4%; 95% CI: 58.1–62.6), underweight women (62.6%; 95% CI: 59.5–65.5), women who had no formal education (64.1%; 95% CI: 62.2–66.0) and those who belonged to the poorest wealth quintile (65.8%; 95% CI: 63.1–68.4). Similarly, the prevalence of anaemia was high among women residing in rural areas (61.5%; 95% CI: 60.0–63.0), Muslims (59.9%; 95% CI: 58.1–61.6) and women with six or more children (62.1%; 95% CI: 60.0–64.1). Also, Igbos (64.7%; 95% CI: 62.1–67.2), Fulanis (64.2%; 95% CI: 59.9–68.4) and Hausa (61.0%; 95% CI: 58.6–61.6) ethnic groups had higher prevalence of anaemia compared to women who are Yoruba or from other ethnic minorities. Generally, the prevalence of anaemia was higher in the south relative to the north except in the South-West. About 7 in 10 women in Sokoto (74.1%; 95% CI: 66.5–80.5), Zamfara (71.6; 95% CI: 65.6–76.8), Katsina (71.1; 95% CI: 64.9–76.6) as well as Anambra (70.5%; 95% CI: 64.4–75.7) and Ebonyi (72.1; 95% CI: 66.8–76.8) were anaemic, while Adamawa (36.0; 95% CI: 30.9–41.5) had the lowest prevalence of anaemia [Fig. 1].

Only 29 (0.2%) women smoked cigarettes, and about 2% had a short stature. For the food taken 24 h before the survey, as presented in Table 2; only 16.6% took eggs and 71.8% of women took meat, fish or poultry and slightly more than half (55%) of women took the minimally acceptable diet. Women who did not take the minimum adequate diet (56.0%; 95% CI: 54.5–57.6) had a lower prevalence of anaemia. Specifically, the prevalence of anaemia was higher among women who did not take: meat, fish or poultry (60.6%; 95% CI: 58.6–62.7), eggs (58.8%; 95% CI: 57.5–60.0), and other fruits (58.8%; 95% CI: 57.4–60.2).

**Table 2:** Dietary characteristics, prevalence and crude risk ratio of women characteristics, NDHS 2018 (N = 14,454)

Variables	Frequency (%)	Prevalence, % (95% CI) p value	Crude RR (95% CI) p value
**Took food made from**			
*Grains, white tubers and roots*		p = 0.045	p = 0.054
No	276 (1.9%)	49.7 (41.7–57.8)	Reference
Yes	14,178 (98.1%)	58.0 (56.8–59.2)	1.06 (1.00–1.11)*
*Legumes*		P = 0.476	p = 0.476
No	7411 (51.3%)	58.2 (56.6–59.8)	Reference
Yes	7043 (48.7%)	57.4 (55.8–59.0)	0.99 (0.98–1.01)
*Nuts and seeds*		p = 0.923	p = 0.923
No	10,041 (69.5%)	57.8 (56.5–59.1)	Reference
Yes	4413 (30.5%)	57.9 (56.0–59.8)	1.00 (0.99–1.01)
*Milk, cheese or yoghurt*		p = 0.188	p = 0.192
No	11,584 (80.1%)	58.2 (56.9–59.5)	Reference
Yes	2870 (19.9%)	56.3 (53.7–58.9)	0.99 (0.97–1.01)
*Meat, fish, poultry*		p < 0.001	p < 0.001
No	4082 (28.2%)	60.6 (58.6–62.7)	Reference
Yes	10,372 (71.8%)	56.7 (55.4–58.0)	0.98 (0.96–0.99)
*Eggs*		p < 0.001	p < 0.001
No	12,061 (83.4%)	58.8 (57.5–60.0)	Reference
Yes	2393 (16.6%)	53.0 (50.4–55.6)	0.96 (0.95–0.98)
*Dark green leafy vegetables*		p = 0.980	p = 0.980
No	3927 (27.2%)	57.8 (55.6–60.0)	Reference
Yes	10,527 (72.8%)	57.8 (56.6–59.1)	1.00 (0.99–1.01)
*Fruits and vegetables rich in Vitamin A*		p = 0.242	p = 0.244
No	9955 (68.9%)	58.2 (56.9–59.6)	Reference
Yes	4499 (31.1%)	56.9 (55.0–58.8)	0.99 (0.98–1.01)
*Other vegetables*		0.052	0.051
No	5357 (37.1%)	59.3 (57.4–61.1)	Reference
Yes	9097 (62.9%)	57.0 (55.6–58.4)	0.99 (0.97–1.00)*
*Other fruits*		p < 0.05	p < 0.05
No	9399 (65.0%)	58.8 (57.4–60.2)	Reference
Yes	5055 (35.0%)	56.1 (54.2–57.9)	0.98 (0.97–0.99)**
*Minimum adequate diet*		p < 0.001	p < 0.001
No	6498 (45.0%)	60.1 (58.4–61.7)	Reference
Yes	7956 (55.0%)	56.0 (54.5–57.6)	0.97 (0.96–0.99)***

*p value < 0.1, **p value < 0.05, ***p value < 0.01

For the house condition as presented in Table 3, only 13.3% of women used clean fuel for cooking, and 56.5% used improved toilet facilities, while 74.8% of women had access to an improved source of water and about 3 in 5 lived in houses with improved floor, wall and roof (Table 3). Women in households that used polluted cooking fuel (59.7%; 95% CI: 58.5–60.9), unimproved source of water (61.5%; 95% CI: 59.4–63.6), unimproved toilet facilities (62.2%; 95% CI: 60.5–63.7) and all unimproved sources of housing materials had a higher prevalence of anaemia. There was a linear relationship between the prevalence of anaemia, wealth quintiles and house status. Women who lived in the least improved house had a higher prevalence of anaemia.

**Table 3:** Social and housing condition, prevalence and crude risk ratio of women characteristics, NDHS 2018 (N = 14,454)

Variables	Frequency (%)	Prevalence, % (95% CI) p value	Crude RR (95% CI) p value
*Cooking fuel*		p < 0.001	p < 0.001
Clean	1927 (13.3%)	46.0 (42.6–49.3)	Reference
Polluted (Mixed/Biomass)	12,527 (87%)	59.7 (58.5–60.9)	1.09 (1.07–1.12)
*Source of water*		p < 0.001	p < 0.001
Unimproved	3637 (25.2%)	61.5 (59.4–63.6)	Reference
Improved	10,817 (74.8%)	56.6 (55.2–58.0)	0.97 (0.95–0.99)***
*Type of toilet facilities*		p < 0.001	p < 0.001
Unimproved	6281 (43.5%)	62.2 (60.5–63.7)	Reference
Improved	8173 (56.5%)	54.5 (52.9–56.1)	0.95 (0.94–0.97)***
*Main floor material*		p < 0.001	p < 0.001
Unimproved	3764 (26.0%)	61.7 (59.5–63.9)	Reference
Improved	10,690 (74.0%)	56.5 (55.1–57.8)	0.97 (0.95–0.98)***
*Main wall material*		p < 0.001	p < 0.001
Unimproved	4333 (30.0%)	63.6 (61.6–65.5)	Reference
Improved	10,121 (70.0%)	55.4 (54.0–56.8)	0.95 (0.94–0.96)***
*Main roof material*		p < 0.001	p < 0.001
Unimproved	1507 (10.4%)	63.8 (60.4–67.1)	Reference
Improved	12,947 (89.6%)	57.1 (55.9–58.4)	0.96 (0.94–0.98)***
*Housing material*		p < 0.001	p < 0.001
Unimproved	1124 (7.8%)	67.0 (63.5–70.2)	Reference
Partially improved	4356 (30.1%)	60.6 (58.5–62.6)	0.96 (0.94–0.98)**
Totally improved	8974 (62.1%)	55.4 (53.8–56.9)	0.93 (0.91–0.95)***

*p value < 0.1, **p value < 0.05, ***p value < 0.01

### Unadjusted and adjusted Poisson model

In the unadjusted model, pregnant women were 3% and those breastfeeding were 2% more likely to have anaemia than other women [Table 1]. The risk of anaemia was higher among women who reside in the rural areas (RR = 1.05; 95% CI: 1.04–1.07), and who were Muslims (RR = 1.02; 95% CI: 1.00–1.04). However, the risk of anaemia reduces with increasing wealth status and level of education. Similarly, a lower risk of anaemia was observed among women aged 20–29 (RR = 0.97; 95% CI: 0.95–0.99) relative to adolescent women and among overweight (RR = 0.96; 95% CI: 0.94–0.97) and obese (RR = 0.92; 95% CI: 0.89–0.94) women relative to those with normal BMI. Also, there was a lower risk of anaemia in women who reported taking minimum adequate diet rich in micronutrient (RR = 0.97; 99% CI: 0.96–0.99) than others (Table 2). Women that lived in houses with partially (RR = 0.96; 95% CI: 0.94–0.98) or totally (RR = 0.93; 95% CI: 0.91–0.95) improved materials, and those from Yoruba or other ethnic minorities had a lower risk of anaemia [Tables 1 and 3].

In the final adjusted model (Model 3, Table 4, Additional file 1), the risk of anaemia was lower in women who took minimum adequate diet (RR = 0.98; 95% CI: 0.97–1.00), those aged 20–24 years relative to adolescent, overweight (RR = 0.97; 95% CI: 0.95–0.99) or obese (RR = 0.93; 95% CI: 0.91–0.96) women compared to those with normal weight. There was a significant inverse relationship between educational status and risk of anaemia, and women who belong to the richer and richest wealth categories were 4% less likely to be anaemic compared to those in the poorest quintile. However, women who were pregnant (RR = 1.03; 95% CI: 0.97–1.00), who reside in rural areas (RR = 1.02; 95% CI: 1.01–1.04), who belong to other Christian denomination (RR = 1.03; 95% CI: 1.01–1.05), who were Muslims (RR = 1.07; 95% CI: 1.04–1.10) and of Igbo ethnic groups (RR = 1.06; 95% CI: 1.01–1.12) were more likely to have anaemia than others. All the selected housing conditions variables were not significantly associated with risk of anaemia with the exception of the type of cooking fuel used in the household. Women who used polluted fuel (RR = 1.04; 95% CI: 1.01–1.07) were more likely to have anaemia compared to those who used clean fuel. Women who were from the North-East were 3% and North-West were 5% less likely to have anaemia, while those in the South-East 6% and South–South were 9% more likely to have anaemia.

**Table 4:** Hierarchical Poisson regression model of factors associated with anaemia

Variables	Model 1	Model 2	Model 3
*Minimum Adequate diet*			
No	Reference (1.0)	Reference (1.0)	Reference (1.0)
Yes	0.97 (0.96–0.99)***	0.98 (0.97–1.00)**	0.98 (0.97–0.99)**
*Woman status*			
other women		Reference (1.0)	Reference (1.0)
Pregnant		1.03 (1.00–1.05)**	1.03 (1.00–1.05)**
Breastfeeding		1.01 (0.99–1.02)	1.01 (0.99–1.02)
*Age*			
15–19		Reference (1.0)	Reference (1.0)
20–24		0.97 (0.95–0.99)**	0.97 (0.95–0.99)**
25–29		0.98 (0.95–1.00)*	0.98 (0.95–1.01)
30–34		1.00 (0.97–1.02)	1.00 (0.97–1.03)
35–39		1.00 (0.97–1.03)	1.00 (0.97–1.03)
40–44		0.99 (0.96–1.02)	0.99 (0.96–1.02)
45–49		0.99 (0.96–1.02)	0.99 (0.96–1.02)
*BMI (kg/m*^*2*^*)*			
< 18.5		1.01 (0.99–1.03)	1.01 (0.99–1.03)
18.5–24.9		Reference (1.0)	Reference (1.0)
25.0–29.9		0.96 (0.95–0.98)***	0.97 (0.95–0.99)***
≥ 30.0		0.93 (0.91–0.96)***	0.93 (0.91–0.96)***
*Parity*			
None		Reference (1.0)	Reference (1.0)
1		1.01 (0.99–1.04)	1.01 (0.99–1.04)
2–3		1.00 (0.98–1.03)	1.00 (0.98–1.03)
4–5		1.00 (0.97–1.03)	1.00 (0.97–1.03)
≥ 6		1.01 (0.98–1.04)	1.01 (0.98–1.04)
*Highest level of education*			
No education		Reference (1.0)	Reference (1.0)
Primary		0.97 (0.95–0.99)**	0.98 (0.96–0.99)**
Secondary		0.96 (0.94–0.98)**	0.97 (0.94–0.99)**
Tertiary		0.94 (0.91–0 .97)***	0.95 (0.92–0.98)**
*Wealth quintiles*			
Poorest		Reference (1.0)	Reference (1.0)
Poorer		0.99(0.97–1.01)	0.99 (0.97–1.02)
Middle		0.98(0.95–0.99)**	0.98 (0.95–1.01)
Richer		0.96(0.93–0.98)***	0.96 (0.93–0.99)**
Richest		0.95 (0.92–0.97)***	0.96 (0.92–0.99)**
*Residence*			
Urban		Reference (1.0)	Reference (1.0)
Rural		1.02 (1.01–1.04)**	1.02 (1.01–1.04)**
*Religion*			
Catholic		Reference (1.0)	Reference (1.0)
Other Christians		1.03(1.01–1.04)**	1.03 (1.01–1.05)**
Islam		1.07 (1.0–1.08)***	1.07 (1.04–1.10)**
Others		0.96 (0.88–1.05)	0.95 (0.88–1.04)
*Ethnicity*			
Fulani		Reference (1.0)	Reference (1.0)
Hausa		1.01 (0.98–1.04)	1.01 (0.98–1.04)
Igbo		1.06 (1.00–1.12)**	1.06 (1.01–1.12)**
Yoruba		0.99 (0.95–1.03)	0.99 (0.95–1.04)
Other ethnic minorities		0.97 (0.94–1.00)*	0.97 (0.94–1.00)*
*Region*			
North Central		Reference (1.0)	Reference (1.0)
North-East		0.97 (0.94–0.99)**	0.97 (0.94–0.99)**
North-West		0.94 (0.92–0.97)***	0.95 (0.92–0.97)***
South-East		1.06 (1.02–1.11)**	1.06 (1.02–1.11)**
South–South		1.09 (1.07–1.12)***	1.09 (1.07–1.12)***
South-West		1.01 (0.98–1.04)	1.02 (0.98–1.05)
*Cooking fuel*			
Clean			Reference (1.0)
Polluted (Mixed/Biomass)			1.04 (1.01–1.07)**
*Source of water*			
Unimproved			Reference (1.0)
Improved			1.01 (0.99–1.03)
*Type of toilet facilities*			
Unimproved			Reference (1.0)
Improved			0.99 (0.97–1.00)
*Housing material*			
Unimproved			Reference (1.0)
Partially improved			0.99 (0.96–1.02)
Totally improved			1.00 (0.96–1.03)

*p value < 0.1, **p value < 0.05, ***p value < 0.01

**Fig. 2:**
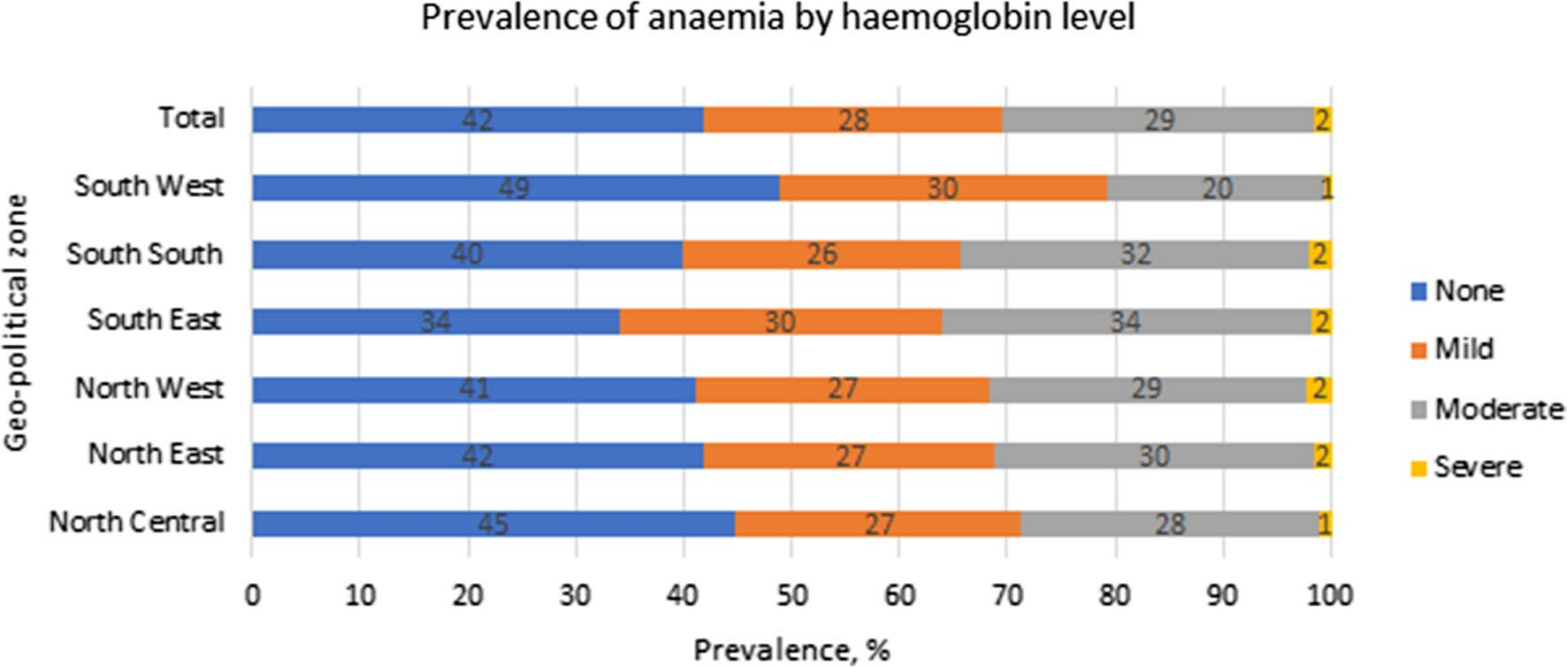
The prevalence of anaemia severity by geopolitical regions

The spatial map revealed that anaemia was highest (at least seven in 10 women) in Sokoto, Katsina and Zamfara in the North-West and Ebonyi in the South–South as well as Anambra in the South-East region of the country [Fig. 1]. Similarly, women in the South-Eastern region had the highest mild (30%) to moderate (34%) forms of anaemia [Fig. 2].

## Discussion

This study investigated the prevalence and factors associated with anaemia using a nationally representative sample of women of reproductive age group. The study showed that more than half (57.8%) of supposed healthy women in Nigerian population were anaemic. There was relatively higher prevalence of anaemia among pregnant women, adolescent (15–19 years), the underweight, women without formal education, poorest women, rural residents and Muslims, as well as women with six or more children. Previous studies in Nigeria [31, 32], Ethiopia [33, 34] and Nepal [35] had similar results.

Like previous studies, we observed high prevalence of anaemia among the poor women, rural residents and women without formal education. In addition, we also found that higher educational attainment, higher wealth and residing in relatively advantageous settings sometimes empower women [36, 37]. A woman that is empowered is more likely to have better access to the right information relative to women that are not economically empowered. Generally, women who are economically empowered are more likely to afford good nutrition and medical care, which in turn reduces the likelihood of their being anaemic [38, 39]. Unlike urban residents, women in rural areas may have difficulty in accessing healthcare facilities due to distant location of such facilities; meanwhile, health facilities are the dominant outlets for anaemia check-up, health education and several other anti-anaemia services [40, 41].

As described in the three delays model, long distances to the health facilities could compromise healthcare utilization [42]. Although we did not consider the distance from participants houses to health facilities in this analysis, it is a common knowledge that healthcare facilities are not within 5 km, which is against one of the key recommendations to ensuring universal access. Currently, Nigerian government is investing in revitalization of primary healthcare to provide basic healthcare including maternal and newborn care [43]. When this programme is fully implemented, it is expected that interventions that will promote behavioural change communication, prevent and promote prompt diagnosis of anaemia as part of basic healthcare package.

Another important risk factors which has been consistently associated with anaemia is inadequate or poor nutrition [23]. Our study showed that Nigerian women with poor nutrition suffered from anaemia. There are multiple dimensions to explain the role of nutrition in anaemia. Poor nutrition could be due to lack of money to prepare or buy food with minimally acceptable nutrient. Sociocultural taboos may discourage women from taking highly nutritious food, and there is a possibility of drug interactions with food, which could limit absorption. It is also plausible that women with chronic medical disorders may suffer from chronic malabsorption. For example, there are some cultural restrictions in Ethiopia that forbids pregnant women with anaemia from taking certain nutritious food and fruits that further worsen their health condition[44]. It is important that relevant agencies of government formulate policy and programmes that discourage perpetuation of sociocultural norms that discourages healthy diet for women. It is equally imperative that government and other stakeholders invest in mass mobilization through culturally acceptable platform to educate people on prevention and timely treatment of anaemia in Nigeria. There should be information through media to educate people on minimum optimal nutritional requirements and dispel misconceptions/myth against food that are rich in adequate nutrients. In essence, women should be motivated to increase fruit and vegetable intake and, also, encourage them to take cheap local diets that can increase their dietary diversity score in order to prevent anaemia [33, 44, 45].

Housing is one of the key indicators of socio-economic status [33, 45]. It is not surprising that previous studies and our study showed a higher prevalence of anaemia in women that lived in poor housing condition. In addition, anaemia was also more common in households that used polluted cooking fuel, unimproved source of water, unimproved toilet facilities and unimproved source of housing materials. Lack of access to basic domestic infrastructure could be an indication of lack of affluence which may negatively affect access to food [44]. The observed variation in the prevalence of anaemia by ethnicity and geographical location in this data might not be direct. It is plausible that there are other underpinning factors such as sociocultural beliefs, interpretations and dispositions to nutrition- as well as specific health-related interventions on nutrition for women at the state and local government levels [46]. For example, there are some states in the Northern Nigeria that are being supported by the World Bank on nutrition during the 2018 NDHS [47]. A qualitative study might further explain the authenticity of the observed diversity or otherwise of the relationship between anaemia and ethnicity or geographical location.

The relatively high prevalence and risk of anaemia among adolescents (15–19 years) relative to adults are well known. The final phase of adolescence is generally characterized with rapid growth and extensive iron requirements [48, 49]. Adolescents gain about 30% weight and at least 20% height during this period with consequent demand for macro- and micronutrients [50]. Other studies from Turkey [51] and Ethiopia [48, 49] have reported the same observation of a relatively higher anaemia among adolescents. These findings underscore the importance of adolescent friendly health and nutrition programmes across health facilities and clinics/infirmaries as a key intervention to minimize the burden of anaemia in this age group.

### Strengths and limitations of the study

This study provides the largest single nationally representative dataset on anaemia among women in West African sub-region. This analysis used other explanatory variables that have not been commonly considered but important in understanding the complexity associated with the burden of anaemia in a low middle-income country. In addition, we used a robust statistical analysis to investigate factors associated with risk of anaemia. Our assessment of anaemia was not based on self-reports, instead, an on-site haemoglobin analysis using a HemoCue®Hb 201 + device. HemoCue has been validated with standard haematocrit estimation process in the laboratory [52]. There are some potential limitations in this study. We were unable to establish a causal relationship between the explanatory variables and anaemia because of the cross-sectional design of the NDHS data used. The dietary and housing factors used in this analysis were not exhaustive. The survey assumed that participants were healthy, but it is plausible that some may have underlying medical condition that could have predisposed them or contributed to the anaemia.

## Conclusion

This study showed that anaemia remained highly prevalent among women of reproductive age in Nigeria. We highlighted the significant role of demographic, nutritional deficiency and lack of basic housing infrastructure as key drivers for anaemia in Nigerian women. We recommend that policy makers and programmers as well as clinicians should design projects that will robustly profile women at risk of anaemia and offer a holistic intervention in preventing and managing anaemia, particularly in high risk groups. It is desirable that researchers should focus on social science research in order to further understand the contextual risks associated with anaemia in women.

## Supplementary Information

**Additional file 1**. **Table S1**. Poisson regression model of factors associated with Mild and Moderate/Severe Anaemia.

## Data Availability

The dataset used for this study can be downloaded after due permission from the DHS Program Archive and can be accessed from https://www.dhsprogram.com/data/dataset/Nigeria_Standard-DHS_2018.cfm?flag=1.
