# Supplementary material for: Ecological analysis of demographic-, nutritional- and housing-related factors associated with anaemia among women of reproductive age group in Nigeria
Source: J Health Popul Nutr. 2022 Dec 9;41:56. doi: 10.1186/s41043-022-00334-6 (PMC9733233; doi:10.1186/s41043-022-00334-6)
Supplement: Supplementary file 1 — Additional file 1. Table S1. Poisson regression model of factors associated with Mild and Moderate/Severe Anaemia. [file 41043_2022_334_MOESM1_ESM.docx]

Supplementary Table 1. Poisson regression model of factors associated with Mild and Moderate/Severe Anaemia

| Model | Crude Poisson | Multivariable Hierarchical Poisson | | |
| --- | --- | --- | --- | --- |
| Variables | Univariate | Model 1 | Model 2 | Model 3 |
| **Adequate micronutrient** |  |  |  |  |
| No | Reference (1.0) | Reference (1.0) | Reference (1.0) | Reference (1.0) |
| Yes | 0.99 (0.98-1.01) | 0.99 (0.98-1.01) | 1.00 (0.99-1.02) | 1.00 (0.98-1.02) |
| **woman status** |  |  |  |  |
| Non-pregnant and non-lactating | Reference (1.0) |  | Reference (1.0) | Reference (1.0) |
| Pregnant | 1.05 (1.03-1.08)*** |  | 1.04 (1.01-1.07)** | 1.04 (1.01-1.07)** |
| Breastfeeding | 1.02 (1.00-1.04)** |  | 1.01 (0.98-1.03) | 1.00 (0.98-1.03) |
| **Age** |  |  |  |  |
| 15-19 | Reference (1.0) |  | Reference (1.0) | Reference (1.0) |
| 20-24 | 1.02 (0.99-1.05) |  | 1.01 (0.98-1.05) | 1.01 (0.98-1.05) |
| 25-29 | 1.03 (1.00-1.06)* |  | 1.03 (0.99-1.07) | 1.03 (0.99-1.07) |
| 30-34 | 1.02 (0.99-1.06) |  | 1.03 (0.99-1.08) | 1.03 (0.99-1.07) |
| 35-39 | 1.02 (0.99-1.05) |  | 1.04 (0.99-1.08)* | 1.04 (0.99-1.08) |
| 40-44 | 1.02 (0.98-1.05) |  | 1.03 (0.99-1.08) | 1.03 (0.99-1.08) |
| 45-49 | 1.00 (0.97-1.04) |  | 1.02 (0.97-1.08) | 1.02 (0.97-1.07) |
| **BMI (kg/m^2^)** |  |  |  |  |
| 18.5-24.9 | Reference (1.0) |  | Reference (1.0) | Reference (1.0) |
| <18.5 | 1.03 (1.00-1.06)** |  | 1.04 (1.01-1.07)** | 1.04 (1.01-1.06)** |
| 25.0-29.9 | 0.99 (0.97-1.02) |  | 1.00 (0.97-1.02) | 1.00 (0.97-1.02) |
| ≥30.0 | 0.93 (0.90-0.96)*** |  | 0.94 (0.91-0.97)*** | 0.94 (0.91-0.97)*** |
| **Parity** |  |  |  |  |
| None | Reference (1.0) |  | Reference (1.0) | Reference (1.0) |
| 1 | 1.02 (0.99-1.05) |  | 1.01 (0.98-1.05) | 1.01 (0.98-1.05) |
| 2-3 | 1.03 (1.00-1.05)** |  | 1.02 (0.98-1.05) | 1.02 (0.98-1.05) |
| 4-5 | 1.03 (1.00-1.06)** |  | 1.01 (0.97-1.05) | 1.01 (0.97-1.05) |
| ≥6 | 1.03 (1.00-1.05)** |  | 0.99 (0.95-1.03) | 0.99 (0.95-1.03) |
| **Highest level of education** |  |  |  |  |
| No education | Reference (1.0) |  | Reference (1.0) | Reference (1.0) |
| Primary | 0.97 (0.94-0.99)** |  | 0.98 (0.95-1.01) | 0.98 (0.95-1.01) |
| Secondary | 0.96 (0.94-0.98)*** |  | 0.98 (0.95-1.01) | 0.98 (0.95-1.01) |
| Tertiary | 0.91 (0.88-0.95)*** |  | 0.95 (0.91-1.00)** | 0.95 (0.91-1.00)** |
| **Wealth quintiles** |  |  |  |  |
| Middle | Reference (1.0) |  | Reference (1.0) | Reference (1.0) |
| Poorest | 1.05 (1.02-1.07)*** |  | 1.04 (1.01-1.07)** | 1.04 (1.01-1.07)** |
| Poorer | 1.03 (1.00-1.06)** |  | 1.03 (1.00-1.05)* | 1.03 (1.00-1.05)* |
| Richer | 1.01 (0.98-1.03) |  | 1.02 (0.99-1.04) | 1.01 (0.99-1.04) |
| Richest | 0.96 (0.93-0.99)** |  | 0.99 (0.96-1.02) | 0.99 (0.96-1.02) |
| **Residence** |  |  |  |  |
| Urban | Reference (1.0) |  | Reference (1.0) | Reference |
| Rural | 1.04 (1.02-1.06) |  | 1.00 (0.98-1.02) | 1.00 (0.98-1.02) |
| **Religion** |  |  |  |  |
| Catholic | Reference (1.0) |  | Reference (1.0) | Reference (1.0) |
| Other Christians | 1.00 (0.99-1.05) |  | 1.02 (0.99-1.05) | 1.02 (0.99-1.05) |
| Islam | 1.02 (0.99-1.05) |  | 1.05 (1.01-1.09)** | 1.05 (1.01-1.09)** |
| Others | 0.99 (0.89-1.11) |  | 0.98 (0.88-1.09) | 0.98 (0.88-1.09) |
| **Ethnicity** |  |  |  |  |
| Fulani | Reference (1.0) |  | Reference (1.0) | Reference (1.0) |
| Hausa | 0.98 (0.95-1.02) |  | 1.00 (0.97-1.04) | 1.00 (0.97-1.03) |
| Igbo | 0.97 (0.94-1.01)* |  | 0.99 (0.93-1.05) | 0.99 (0.93-1.06) |
| Yoruba | 0.90 (0.87-0.94)*** |  | 0.99 (0.94-1.05) | 0.99 (0.94-1.05) |
| Other ethnic minorities | 0.97 (0.94-1.00)* |  | 0.99 (0.95-1.03) | 0.99 (0.95-1.03) |
| **Region** |  |  |  |  |
| North Central | Reference (1.0) |  | Reference (1.0) | Reference (1.0) |
| North East | 1.01 (0.98-1.04) |  | 0.97 (0.95-1.01) | 0.97 (0.94-1.00)* |
| North West | 1.01 (0.98-1.04) |  | 0.97 (0.94-1.00)* | 0.97 (0.94-1.00)* |
| South East | 1.01 (0.98-1.04) |  | 1.06 (1.00-1.12)** | 1.06 (1.00-1.12)** |
| South South | 1.03 (1.00-1.06)** |  | 1.07 (1.04-1.11)*** | 1.07 (1.04-1.12)*** |
| South West | 0.92 (0.89-0.95)*** |  | 0.94 (0.90-0.99)** | 0.94 (0.90-0.99)** |
| **Cooking fuel** |  |  |  |  |
| Clean | Reference (1.0) |  |  | Reference (1.0) |
| Polluted (Mixed/Biomass) | 1.08 (1.04-1.11)*** |  |  | 1.01 (0.98-1.05) |
| **Source of water** |  |  |  |  |
| Unimproved | Reference (1.0) |  |  | Reference (1.0) |
| Improved | 0.97 (0.95-0.99)** |  |  | 1.00 (0.98-1.02) |
| **Type of toilet facilities** |  |  |  |  |
| Unimproved | Reference (1.0) |  |  | Reference (1.0) |
| Improved | 0.98 (0.96-1.00)** |  |  | 1.02 (1.00-1.04) |
| **Housing material** |  |  |  |  |
| Unimproved | Reference (1.0) |  |  | Reference 1.0 |
| Partially improved | 0.98 (0.96-1.01) |  |  | 1.00 (0.97-1.03) |
| Totally improved | 0.95 (0.92-0.97)*** |  |  | 0.99 (0.95-1.03) |

*p-value <0.1, **p-value <0.05, ***p-value<0.01
